# Comments on: “Asymptomatic abdominal wall and incisional hernias: Is therapeutic decision consensual? An international survey”

**DOI:** 10.1016/j.amsu.2021.102185

**Published:** 2021-03-04

**Authors:** Sujala N.R. Kalipershad

**Affiliations:** Tameside and Glossop Integrated Care NHS Foundation Trust, Tameside General Hospital, Ashton-under-Lyne, Greater Manchester, UK

Dear Editor,

I read the paper by Jaquet et al., on “Asymptomatic Abdominal Wall and Incisional Hernias: Is Therapeutic Decision Consensual? An International Survey”, published in the Annals of Medicine and Surgery in December 2020, with great interest. The topic of asymptomatic hernia repair is particularly noteworthy in the current healthcare climate with the postponement of non-urgent surgery and the exceedingly long waiting list that will follow once the pandemic is better controlled and elective surgery has resumed. Furthermore, this is of interest to trainees in the NHS because of the variation in practice and the need to apply for funding prior to listing a patient for a hernia repair. A justification of impact of the patient's symptoms on their lives is required and funding is not approved for asymptomatic hernias. With the lack of consensual practice comes the uncertainty of what different consultant's practice is. It is worth noting that it is unlikely that we will have consensus enough to have an established guideline or recommendation.

There are a few points within the paper, I feel require further explanations for example the age of the surgeons was used in the analysis, however, there was no definition with regards to grade or experience of the surgeons, neither the relationship between the age and number of hernias performed per month. The scenarios used by the authors were realistic and could be expected in any clinic. The options of abstention and the surgical options are described, however, the option of “I do not retain any indication for operation or watchful waiting” is unclear and unexplained in the body of the paper. With each case there is a percentage of the surgeons surveyed, having chosen abstention or surgery, which is derived from the 6 treatment options for each case, however, in cases 2 to 5 these calculations do not add up.

Within the body of the paper the authors report consensual attitude in case 3 (asymptomatic inguinal hernia in an elderly patient with heavy comorbidities, in favour of surgical abstention) but the percentage for abstention is 69% and the definition of consensual attitude is 75%. It is indeed the following case (case 4 - 55-year-old patient with no major medical-surgical history, referred for the discovery of a small asymptomatic inguinal hernia during a urologist's assessment of benign prostatic hyperplasia) which according to calculations mentioned in Table 1 shows consensual attitude (83%) in favour of abstention. A minor point is that the post-partum patient in case 9 was said to have sought advice again for ‘his’ hernia.

Without the calculations being reviewed and explained, the data within this paper is difficult to analyse and its relatability and impact on practice is uncertain. I would be keen to have these points clarified by the authors and feel that this will enhance my understanding of the paper and its applicability to practice.

## Conflicts of interest

None.

## Sources of funding

None.

## Ethical approval

None.

## Consent

There are no patients involved.

## Author contribution

There is only one author.

## Registration of Research Studies

1. Name of the registry: N/A.

2. Unique Identifying number or registration ID: N/A.

3. Hyperlink to your specific registration (must be publicly accessible and will be checked): N/A.

## Guarantor

Sujala Kalipershad.

